# Glycogen Quantification and Gender Identification in Di-, Tri-, and Tetraploid *Crassostrea gigas* Using Portable Near-Infrared Spectroscopy

**DOI:** 10.3390/foods13142191

**Published:** 2024-07-11

**Authors:** Jingjing Fu, Weijun Wang, Youmei Sun, Yousen Zhang, Qihao Luo, Zhongping Wang, Degang Wang, Yanwei Feng, Xiaohui Xu, Cuiju Cui, Guohua Sun, Zan Li, Jianmin Yang

**Affiliations:** 1 School of Agriculture, Ludong University, Yantai 264025, China; fjj000117@163.com (J.F.); sunyoumei4554@163.com (Y.S.); 19909024486@163.com (Y.Z.); fywzxm1228@163.com (Y.F.); xxh83121@163.com (X.X.); cuicuiju@163.com (C.C.); sgh_smile@163.com (G.S.); lizanlxm@163.com (Z.L.); ladderup@126.com (J.Y.); 2 Yantai Haiyu Marine Technology Co., Ltd., Yantai 264000, China; 3 Yantai Kongtong Island Industrial Co., Ltd., Yantai 264000, China; 17854266371@163.com (Q.L.); ktdsy6690740@126.com (Z.W.); 4 Shandong High-Speed Marine Technology Co., Ltd., Yantai 264003, China; sdgshbcy@163.com

**Keywords:** *Crassostrea gigas*, tetraploid, near-infrared spectroscopy, gender, quantitative model, qualitative model

## Abstract

Near-infrared spectroscopy (NIR) has become an essential tool for non-destructive analysis in various fields, including aquaculture. This study presents a pioneering application of portable NIR spectrometers to analyze glycogen content in the gonadal tissues of the Pacific oyster (*Crassostrea gigas*), marking the first instance of developing quantitative models for glycogen in tetraploid *C. gigas*. The research also provides a comparative analysis with models for diploid and triploid oysters, underscoring the innovative use of portable NIR technology in aquaculture. Two portable NIR spectrometers were employed: the Micro NIR 1700 (908–1676 nm) and the Micro PHAZIR RX (1624–2460 nm). Near-infrared spectra were acquired from the gonadal tissues of diploid, triploid, and tetraploid *C. gigas*. Quantitative models for glycogen content were developed and validated using cross-validation methods. Additionally, qualitative models for different ploidies and genders were established. For the Micro NIR 1700, the cross-validation correlation coefficients (Rcv) and cross-validation relative predictive errors (RPDcv) for glycogen were 0.949 and 3.191 for diploids, 0.915 and 2.498 for triploids, and 0.902 and 2.310 for tetraploids. The Micro PHAZIR RX achieved Rcv and RPDcv values of 0.781 and 2.240 for diploids, 0.839 and 2.504 for triploids, and 0.717 and 1.851 for tetraploids. The Micro NIR 1700 demonstrated superior quantitative performance, with RPD values exceeding 2, indicating its effectiveness in predicting glycogen content across different ploidy levels. Qualitative models showed a performance index of 91.6 for diploid and 95 for tetraploid genders using the Micro NIR 1700, while the Micro PHAZIR RX achieved correct identification rates of 99.79% and 100% for diploid and tetraploid genders, respectively. However, differentiation of ploidies was less successful with both instruments. This study’s originality lies in establishing the first quantitative models for glycogen content in tetraploid *C. gigas* using portable NIR spectrometers, highlighting the significant advancements in non-destructive glycogen analysis. The applicability of these findings is substantial for oyster breeding programs focused on enhancing meat quality traits. These models provide a valuable phenotyping tool for selecting oysters with optimal glycogen content, demonstrating the practical utility of portable NIR technology in aquaculture.

## 1. Introduction

The Pacific oyster (*Crassostrea gigas*) belongs to the phylum Mollusca, class Bivalvia, order Pterioida, family Ostreidae, and is commonly known as the long oyster or cupped oyster. It is the primary oyster species cultivated in the northern coastal areas of China, known for its fast growth, plump meat, and strong resistance to infectious diseases. Traditionally, wild oysters are diploid, but diploid oysters are adversely affected during the breeding season in summer, leading to poor meat quality and high mortality rates, and as a result, they cannot meet the needs of market demands. Genetic improvement of oysters through polyploidization has garnered attention from farmers and researchers worldwide. In 1986, successful induction of triploid *C. gigas* was achieved [1]. Triploid oysters exhibit superior growth performance, particularly during the summer breeding season, when they exhibit almost no reproductive ability. More energy and nutrients are directed toward their own growth and development, resulting in higher glycogen content and significantly greater weight compared to diploid oysters. Moreover, low reproductive ability of triploids also safeguards the natural genetic resources of oyster germplasm [2,3,4]. Currently, the cultivation of triploid oysters is primarily achieved through the hybridization of diploid and tetraploid oysters, or through chemical induction methods [5]. Chemical induction, by disrupting meiotic division I or II and retaining the first or second polar body, has a high likelihood of producing triploid oysters, though with low survival rates and residual drugs [6]. Hybridization of diploid and tetraploid oysters, a biological method, is the optimal approach for producing triploids. Both reciprocal crosses result in triploid offspring, achieving a 100% triploid population. Currently, research on oyster genetic breeding both domestically and internationally has mainly focused on diploid oysters with characters of growth [7,8], shell colors [9,10], and glycogen content [11,12]. So far little has been done on selective breeding of the tetraploid oyster, especially on meat quality traits of high-quality oysters. Therefore, the introduction of novel, fast, and accurate quality-control methods is of great significance for the selective breeding of oysters.

Near-infrared spectroscopy (NIRS), as a modern analytical technique, has demonstrated its advantages in terms of rapid, convenient, and non-destructive detection across various fields, providing an innovative solution for qualitative and quantitative analysis of organic chemical substances [13]. Particularly in the analysis of plant and animal products, NIRS has successfully replaced traditional chemical measurement procedures, offering more efficient analytical methods for industries [14,15,16]. In previous studies, Fluckiger et al. demonstrated the effectiveness of NIRS technology in monitoring abalone quality and quantitatively analyzing glycogen content [17]. For oysters, glycogen serves as a crucial energy storage material, playing a key role in their physiological metabolism [18], and the accurate determination of its content is important for assessing the nutritional value, growth status, and physiological health of oysters. The uniqueness of this study lies in the establishment of a high-throughput glycogen quantitative model for tetraploid *C. gigas* using a portable near-infrared (NIR) spectrometer, as well as the development of glycogen quantitative models for diploid and triploid oysters. Portable NIR spectrometers offer significant advantages, including portability for on-site analysis, minimal sample requirements with no need for complex preparation, non-destructive testing preserving sample integrity, instant results reducing analysis time, and user-friendly operation facilitating use by non-experts. These features make portable NIR spectrometers valuable for real-time process control, quality assurance, and in-field decision-making across agriculture, food safety, pharmaceuticals, chemical industries, and environmental monitoring. They could obtain the glycogen content data in real-time on-site at the breeding facility, not just under laboratory conditions. More importantly, the selected individual oysters can be used as parents for offspring breeding. To achieve qualitative research and identification of male and female oysters, we have also established near-infrared qualitative models for diploid, triploid, and tetraploid oysters, as well as for male and female oysters. This innovation not only provides an efficient means for parent-controlled breeding of high-quality oyster strains but also opens up new possibilities for the development of the oyster aquaculture industry. These models could serve as a scientific basis for selective breeding and quality evaluation research on *C. gigas*, which have the potential to enhance the efficiency of quality trait breeding of oysters and could promote oyster industry sustainable development. 

## 2. Materials and Methods

### 2.1. Sample Collection and Preparation

**Figure 1:**
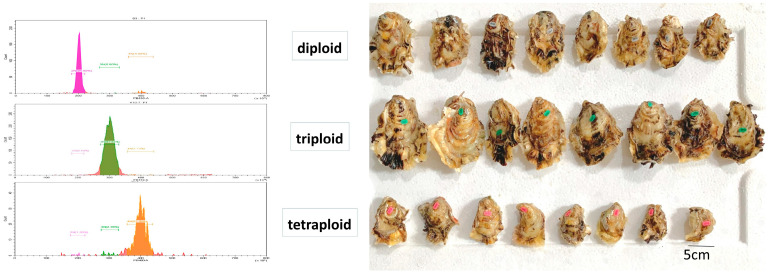
Representative images of the diploid, triploid, and tetraploid *C. gigas* samples.

The samples used in this study were collected from the Kongtong Island Genetic Breeding Center in Yantai City, Shandong Province, China. During breeding season, a total of 679 *C. gigas* samples were collected, including 187 diploids (2N), 147 triploids (3N), and 345 tetraploids (4N). Among them, gonads were fully developed, and there were 122 female and 65 male diploid oysters, and 134 female and 211 male tetraploid oysters. Diploid, triploid, and tetraploid oysters were all derived from the new Pacific oyster strain of Luyi No. 1 (GS-01-006-2020). Representative images of the diploid, triploid, and tetraploid *C. gigas* samples are shown in Figure 1. Fresh oysters were shucked, and the gonadal portion was covered with plastic film for NIR spectrum collection. Spectra of the gonadal portion were collected using the MicroNIR 1700 near-infrared spectrometer (JDSU, Milpitas, CA, USA) and the Micro PHAZIR RX near-infrared spectrometer (Thermo Fisher, Waltham, MA, USA). After collection, a piece of gonad was cut down with a surgical knife at the position that was measured by near-infrared spectrometer, and the cut portions were placed in sealed bags and stored in a −80 °C freezer until subsequent determination of chemical composition. 

### 2.2. Determination of Glycogen Content

The glycogen content of different ploidy oyster samples was analyzed using a glycogen detection kit based on the anthrone method with glucose as the standard (Nanjing Jiancheng Bioengineering Institute, A043-1-1, Nanjing, China). Take 22–23 mg of powdered samples and transfer them into a test tube. Add alkali solution to the sample in a 1:3 ratio, and then heat the mixture in a boiling water bath for 30 min. Subsequently, dilute the hydrolysate to 100 times the weight of the sample. Next, take 100 μL of the hydrolysate, add 2 mL of the coloring reagent, and heat the sample in a boiling water bath for 5 min. Finally, use a microplate reader (Thermo Fisher Scientific, ZnFINITE200PRO, Reinach, Switzerland) to measure the OD value of each sample at 620 nm.

### 2.3. Portable NIR Spectroscopy System and Spectrum Collection

**Figure 2:**
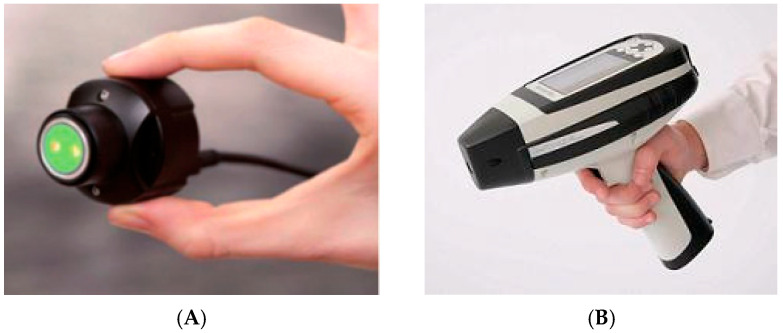
(**A**) MicroNIR 1700 NIR spectrometer. (**B**) Micro PHAZIR RX NIR spectrometer.

To facilitate portable near-infrared spectroscopy research, the MicroNIR 1700 near-infrared spectrometer (MN) and the Micro PHAZIR RX near-infrared spectrometer (MP) were employed for comparison (Figure 2). MN is a miniature system based on a linear variable filter (LVF) monochromator, seamlessly integrating a light source, optical elements, electronic components, and basic operating software. It captures spectra in the wavelength range of 908–1676 nm, targeting the absorption bands of high-energy chemical bonds in organic substances. With a scanning speed of 4 scans/second and dimensions of only 45 × 42 mm, MN is currently one of the smallest near-infrared spectrometers available on the market. MN primarily processes the light source through a grating, and then the detector illuminates the sample signal, ultimately producing near-infrared spectra that vary with wavelength. The near-infrared probe temperature was maintained between 40 and 50 °C during spectrum collection, with each sample accumulating 10 scans. The Unscrambler X 10.4 software was employed to average each oyster sample spectrum for further analysis.

The MP is one of the world’s first handheld NIR analyzers, a NIR miniature spectrometer based on a grating light modulator. It does not require external equipment when collecting spectra, and is a small NIR spectrometer that can be operated independently to truly realize portable NIR analysis. The collected near-infrared wavelength range is 1624–2460 nm, covering the characteristic range of covalent bond overtones and first overtones. However, the resolution of the instrument is relatively low, only 22 nm. The instrument undergoes a 10 min warm-up and self-check before each spectrum collection. To eliminate background effects, a built-in program is used to tune the instrument to spectrum collection mode. Each sample accumulates five scans. Finally, the software of Method Generator which was inbuilt with the instrument is used to select the optimal spectrum for each sample for subsequent analysis.

### 2.4. Spectral Data Preprocessing

Before constructing the model, spectral preprocessing methods were employed to mitigate the adverse effects of external environmental changes, sample density, and temperature variations on spectral information. These methods aimed to eliminate high-frequency random noise, baseline drift, sample inhomogeneity, and other issues.

For MN, a combination of original spectra (SP), first derivative (FD), second derivative (SD), no smoothing (NS), Savitzky–Golay filter (SG), and Norris derivative filter (NDF) were used in preprocessing the spectra and corresponding true values. This approach reduced noise in the spectral information while retaining potential spectral data relevant to chemical information, allowing for the selection of the optimal spectral preprocessing method. Additionally, Mahalanobis distance was employed to identify outliers in the model, leading to the exclusion of certain samples.

For MP, an evaluation was conducted using SP, Baseline Offset (BO), Baseline Linear (BL), Normalize Max (NM), Normalize Range (NR), Normalize Peak (NP), Norm Unit Vector (NUV), Normalize Area (NA), SG, Multiplicative Scatter Correction (MSC), and Standard Normal Variate (SNV). Various preprocessing methods for both instruments were compared to determine which method could effectively enhance the model’s performance.

Based on the chosen preprocessing methods, quantitative models for *C. gigas* diploid, triploid, and tetraploid glycogen content, as well as qualitative models for different ploidy and gender of diploid and tetraploid *C. gigas*, were established using the two portable instruments. The described spectral preprocessing methods were sequentially applied to optimize the near-infrared data across the entire wavelength range.

### 2.5. Model Construction: Calibration and Validation

#### 2.5.1. Qualitative Modeling

In each group of samples, 30 randomly selected samples were used to build qualitative models for differentiating between various ploidies and genders of *C. gigas*. The established models are expected to effectively distinguish between oysters of different ploidies and genders. The qualitative models were created using the decision model provided by chemometrics software. TQ Analyst software (Version 9.8.208) was utilized to process the spectral data collected by the MN (MicroNIR 1700) instrument, employing Discriminant Analysis (DA) with Mahalanobis distance (95% confidence) for the qualitative analysis of ploidy. Performance index (PI) and cumulative contribution rates were used to evaluate the performance of the qualitative model. A high PI value usually means that the model is able to accurately predict the class of samples and also maintains a high level of stability and reliability, with a maximum value of 100, the closer to 100 the better. It is calculated by the following formula:

$$\mathrm{PI}=\frac{\left|actual-calculated\right|}{expected}\times 100$$

The spectra collected by the MR (Micro PHAZIR RX) instrument were processed using the software Method Generator (Version 5.3.0.42), and the Classify function was used for qualitative analysis of the samples. Finally, the unvalidated samples were used for verification, and the verification quality was assessed based on accuracy.

#### 2.5.2. Quantitative Modeling

A total of 679 samples were randomly divided into calibration set and validation set. Partial Least Squares (PLS) regression was chosen as the chemometric method for building the calibration model. The full spectral range collected by the instruments was selected, and the model was optimized and validated. Various spectral preprocessing methods were screened to ensure obtaining the most ideal mathematical model with optimal mathematical indicators. During the modeling process, outliers were removed. Mahalanobis distance was used to eliminate outlier values, and Dixon’s test was employed to remove exceptional spectral data, enhancing the stability and robustness of the model. To avoid overfitting, cross-validation was performed by partitioning three samples each time from the calibration set, and the remaining samples were used for modeling. This process was repeated until all samples were validated, and each sample was verified only once, obtaining cross-predicted values for all samples. External validation was conducted using samples outside the calibration set. To ensure the accuracy and reliability of the model, the validation samples accounted for 1/9 of the total samples. Statistical parameters included Root Mean Square Error of Calibration (RMSE_C_), Calibration Correlation Coefficient (R_C_), Root Mean Square Error of Cross-Validation (RMSE_CV_), Cross-Validation Correlation Coefficient (R_CV_), Root Mean Square Error of Prediction (RMSE_P_), Prediction Correlation Coefficient (R_P_), and Relative Percent Difference (RPD). A good calibration model has an R value closer to 1, and lower RMSE_CV_ and RMSEP values indicate better predictive ability. Higher RPD values reflect better predictive ability. RPD < 1.4 indicates an unreliable model result, 1.4 < RPD < 2.0 suggests a relatively reliable model suitable for practical analysis, and RPD > 2.0 indicates a highly reliable model [19]. The formula is as follows:

$$RMSE=\sqrt{\frac{\sum_{i=1}^{N}{\left(\hat{y_{i}}-y_{i}\right)}^{2}}{N}}$$

$$R^{2}=1-\frac{\sum_{i=1}^{N}{\left(\hat{y_{i}}-y_{i}\right)}^{2}}{\sum_{i=1}^{N}{\left(y_{i}-\bar{y_{i}}\right)}^{2}}$$

$$\mathrm{RPD}=\frac{SD}{RMSE}$$

where $\hat{y_{i}}$ and y_i_ are the measured and predicted values of glycogen content, respectively; N is the number of samples in the modeling or validation set; $\bar{y_{i}}$ is the y_i_ is the mean value; and SD is the standard deviation of the measured values in the validation set. These parameters were used to evaluate the performance of each calibration model related to ploidy-specific *C. gigas* with the trait of glycogen content for predicting glycogen content of unrelated oyster samples.

## 3. Results and Discussion

### 3.1. Descriptive Statistics of Glycogen Content Indicators

After the removal of outliers and internal cross-validation, quantitative models for glycogen in *C. gigas* with different ploidies were constructed based on the near-infrared spectra collected by different instruments. The composition of samples in the calibration set and validation set for the respective models is shown in Table 1. Models constructed from spectra acquired by MP instruments are limited by dedicated software and cannot be externally validated.

**Figure 3:**
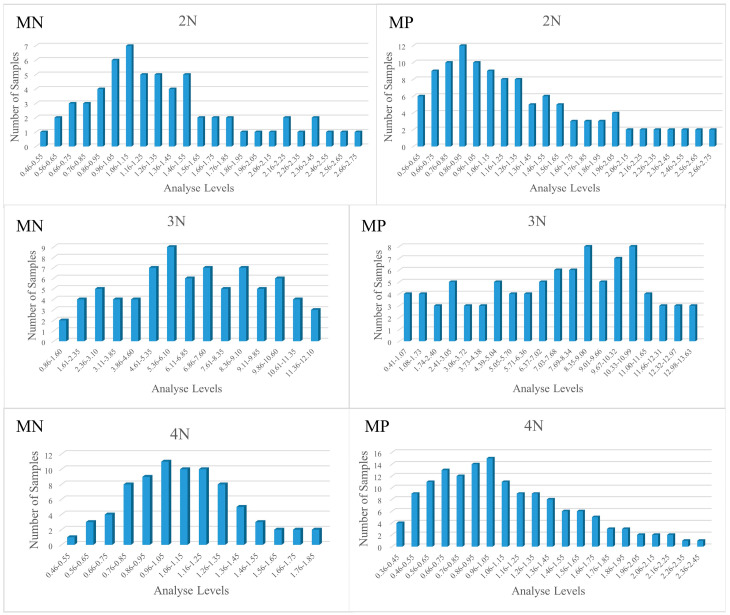
Distribution of Glycogen Content in Different Ploidy *C. gigas* Samples. Note: 2N, 3N, 4N indicate diploid, triploid, tetraploid; MN, MP indicate: MicroNIR 1700 Near-Infrared Spectrometer, Micro PHAZIR RX Near-Infrared Spectrometer.

To ensure representative quantitative results, samples should be distributed as uniformly as possible, and the spectral data should be collected to form a representative sample set. The glycogen content values of different ploidy oyster samples roughly followed the uniformly standard. The distribution of all samples for glycogen content in this study is shown in Figure 3. The sample distribution approximates a normal distribution, meeting the requirements for a wide distribution range of sample contents during the establishment of near-infrared analysis models.

The results indicate that the triploid oysters have a significantly higher glycogen content compared to diploid and tetraploid oysters. This observation aligns with the known physiological differences between triploid and other ploidy levels. Triploid oysters are typically sterile and allocate more energy towards growth and glycogen storage rather than reproduction, as noted by Allen and Downing (1986) in their study on the energetic allocation in triploid oysters [1]. This higher glycogen content is advantageous for meat quality, making triploid oysters a desirable choice in aquaculture, consistent with the findings of Nell (2002) regarding the benefits of triploidy in aquaculture species [20]. The MN instrument demonstrated a consistent ability to quantify glycogen across all ploidy levels, as evidenced by the narrow range of glycogen content and lower standard deviations in its measurements. This suggests that the MN instrument might have better sensitivity and specificity for detecting glycogen in oyster tissues compared to the MP instrument. The broader range of glycogen content detected by the MP instrument in triploid oysters suggests higher variability, which could be attributed to differences in the sample preparation, spectral resolution, or the instrument’s calibration, similar to observations made by Kawano et al. (1993) in their comparative study of NIR spectrometers [21]. The limitations in external validation for the MP instrument highlight the necessity for improved software capabilities to enhance its application in quantitative analysis. The uniform distribution of glycogen content values and the approximation of a normal distribution in the samples ensure that the models developed are robust and reliable. These models can be applied to predict glycogen content in a broad range of oyster samples, which is critical for breeding programs aiming to enhance meat quality traits, as supported by the work of Magwaza et al. (2016) on the importance of robust models in breeding programs [22].

Overall, this study confirms the potential of portable NIR spectrometers in accurately quantifying glycogen content in oysters of different ploidies. The findings provide a valuable reference for the selection and breeding of oysters with superior meat quality, emphasizing the practical applicability of these models in aquaculture. These results are in line with previous studies highlighting the importance of NIR technology in aquaculture applications [23].

### 3.2. Spectral Analysis

**Figure 4:**
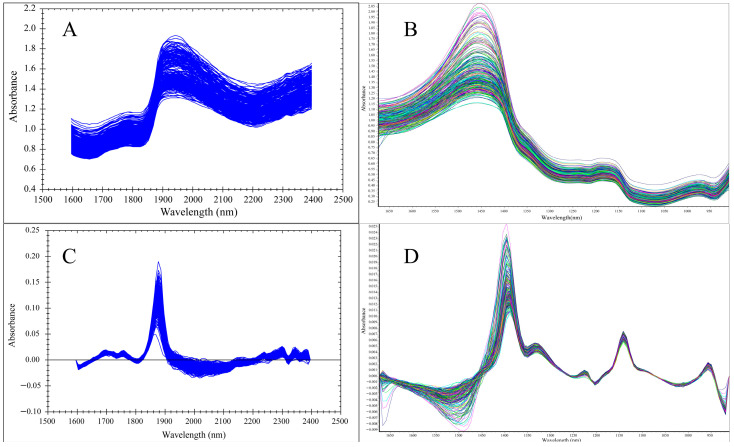
NIR original spectra and first derivative spectra of all *C. gigas* gonad tissue samples (using tetraploid as an example). (**A**) Original spectra from MP. (**B**) Original spectra from MN. (**C**) First derivative spectra from MP. (**D**) First derivative spectra from MN.

The raw NIR spectra of all oyster samples were collected under different instruments, using the spectrum of tetraploid oysters as an example (Figure 4). The obtained NIR spectra exhibit overtones and combination bands of hydrogen-containing groups (-OH, -NH, and -OH vibrations) in the oyster gonad. Comparison of raw and first-order derivative spectra which were collected by different NIR spectrometers, using the tetraploid oyster as an example (Figure 4). The rich spectral information provides extensive insights for both quantitative and qualitative analysis of *C. gigas* using NIR. 

The NIR spectra of oyster gonads display characteristic absorption peaks that are critical for identifying and quantifying glycogen content. The prominent absorption peaks between 1400 and 1500 nm and 1800 and 2000 nm are indicative of the biochemical composition of the oyster tissue. Specifically, the 1400–1500 nm region corresponds to the first overtone of O–H stretching vibrations, which is a common feature in water and other hydroxyl-containing compounds [24]. The 1800–2000 nm region represents combination bands of O–H stretching and deformation vibrations, providing additional information about the hydrogen-bonded structures within the tissue, corroborating findings from Osborne et al. (1993) on the interpretation of NIR spectra [25]. The comparison of raw and first-order derivative spectra highlights the advantages of derivative preprocessing in enhancing spectral features and clarifying peak identification. The findings underscore the importance of selecting appropriate spectral preprocessing methods to enhance the quality of the spectral data.

### 3.3. Spectral Data Preprocessing

To identify the optimal spectral preprocessing method, we compared various method combinations. When quantitative models were established, we assessed model quality using prediction errors and correlation coefficient. The highest correlation coefficient and lowest prediction error indicated the best preprocessing method. For qualitative model construction, we applied an evaluation standard of correct identification rates, employing different preprocessing methods for the three ploidy types in the NIR models. Based on diverse preprocessing approaches, we established glycogen models for the different ploidy types of *C. gigas* gonad tissues under different NIR spectral instruments, individually. The analysis showed that preprocessing methods significantly impact model performance. For quantitative models, methods that effectively reduced noise and baseline drifts resulted in higher determination coefficients and lower prediction errors, indicating more precise glycogen content predictions in *C. gigas* gonad tissues. This finding aligns with the work of Rinnan et al. (2009), who highlighted the importance of preprocessing in enhancing the accuracy of NIR models [26]. Simultaneously, we developed qualitative models for different genders and the ploidy types of diploid and tetraploid. Following preprocessing across the entire spectral range, we utilized various principal component numbers to establish principal component analysis models. The optimal preprocessing method and principal component number were determined based on the model’s correct identification rate, similar to the approach used by Berrueta et al. (2007) for multivariate calibration [27]. The detailed information in Table 2, Table 3 and Table 4, which outline the preprocessing methods and principal component numbers, provides valuable insights for replicating and extending this work. By refining spectral preprocessing techniques, future studies can further enhance the applicability and precision of NIR spectroscopy in oyster breeding programs, ultimately aiding in the selection of oysters with high glycogen content.

### 3.4. Qualitative Model Construction

**Figure 5:**
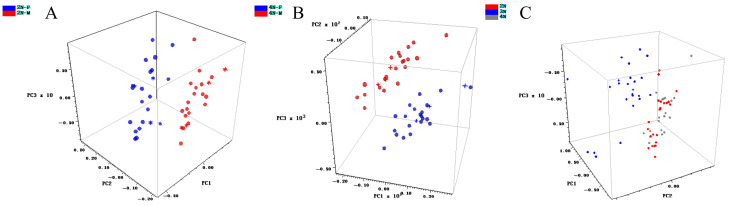
Principal component three-dimensional score plots of the MN instrument for samples with different ploidies and genders. (**A**) Tetraploid gender qualitative. (**B**) Diploid gender qualitative. (**C**) Qualitative analysis of different ploidies. Note: The * and + in the image indicate validation sets.

The spectra collected by the MN instrument were subjected to qualitative analysis using TQ software to assess the gender and ploidy of *C. gigas* diploid and tetraploid individuals. Discriminant analysis was employed to establish principal component analysis (PCA) models for 2N, 3N, and 4N, as well as 2N-M, 2N-F, 4N-M, and 4N-F oysters after preprocessing the near-infrared spectra. The results showed that the diploid and tetraploid models correctly identified without misclassifications (Table 3). The cumulative contribution rate of the 10th principal component exceeded 99%, indicating that the data collected in the range of 908–1676 nm could fully explain the characteristic information of oyster samples. Subsequently, PCA was used to establish a near-infrared classification model for *C. gigas*, enabling the classification and identification of its ploidy and gender. When PCA was used for data analysis, it could express data in low-dimensional space from high-dimensional space [28]. By reducing the dimensionality of data through PCA, a few new variables can be used to replace original variables, reflecting the characteristic information of the original variables as much as possible. The three-dimensional score plot of principal components for samples with different ploidies and genders was shown in the figure (Figure 5). The horizontal axis represents the score values of PC1, and the vertical axis represents the score values of PC2, intuitively displaying the separation between different samples. There were significant differences in the information load of the three models on PC1 and PC2. Diploid and tetraploid male and female oysters could be well distinguished in the principal component space, exhibiting excellent clustering and complete correct sample differentiation, especially with good separation for different ploidies. The models were validated, and performance parameters are shown in Table 3. The performance index of the diploid gender qualitative model was 91.6, that of the tetraploid gender model was 95, and that of the different ploidy model was 83.1. The results of qualitative modelling of different ploidies are not sufficient to make accurate judgements.

**Figure 6:**
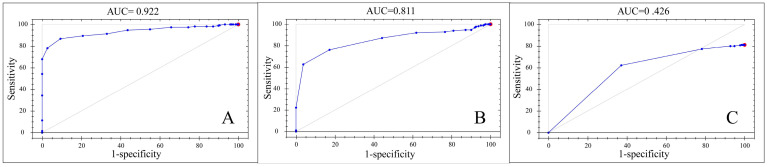
ROC curves of the MP instrument for samples with different ploidies and genders. (**A**) Diploid gender qualitative. (**B**) Tetraploid gender qualitative. (**C**) Qualitative analysis of different ploidies.

Spectra collected by the MP instrument were subjected to qualitative analysis using the Classify module in the Method Generator software. As shown in the figure (Figure 4), the diploid and tetraploid gender qualitative models could well distinguish between males and females, with correct recognition rates of 100%. However, the correct recognition rate of the different ploidy qualitative model was 81.17%, indicating that it could not separate the three types of samples effectively. The performance of the qualitative models of the MP instrument was assessed using subject operating characteristic (ROC) curves, and the ROC curves for the three models are shown in Figure 6. The curves demonstrate the relationship between sensitivity and specificity. In the figure, the x-axis represents specificity, while the y-axis represents sensitivity. The closer the ROC curve is to the upper left corner, the larger the area covered underneath it, which indicates that the model is more discriminative and able to distinguish between different samples more effectively. An AUC value close to 1 indicates higher predictive model accuracy. When AUC > 0.7, the model performance is considered good [29,30]. As shown in Table 4, the AUC value of the diploid gender qualitative model is 0.922, and that of the tetraploid model is 0.811, indicating that the model can well distinguish between males and females. However, the AUC value of the different ploidy qualitative model is only 0.426, indicating poor model performance and an inability to distinguish between different ploidies of *C. gigas*. The research results indicate that both instruments cannot distinguish well between oysters with different ploidies. Hawkins et al. demonstrated that the performance of triploid *C. gigas* in terms of growth, feeding rate, and absorption efficiency is superior to that of diploids, attributing these results to triploid oysters being partially or completely sterile, with all the energy otherwise used for reproduction allocated to the growth of tissues and shells [31], while the fertility of tetraploids with reproductive capability and diploids may result in their physiological similarities. Haure et al. demonstrated that the growth rate of tetraploids is comparable to that of triploids and higher than that of diploids [32]. Thus, the heterozygosity of triploid and tetraploid *C. gigas* may contribute to their similar growth rates. These reasons may lead to possible overlap of spectral features between different ploidy levels when using spectral analysis instruments, and this similarity may affect the resolution of spectral analyses by giving them similar absorption or reflectance properties in certain spectral regions, making it difficult for models to differentiate between different ploidy oysters in a given wavelength range. Accurately distinguishing the gender and ploidy of oysters can provide a scientific basis for genetic improvement and breeding of oysters, thus optimizing the production process and increasing the economic value of oysters.

### 3.5. Quantitative Model Construction and Optimization

#### 3.5.1. Instrument Performance and Model Comparison

**Figure 7:**
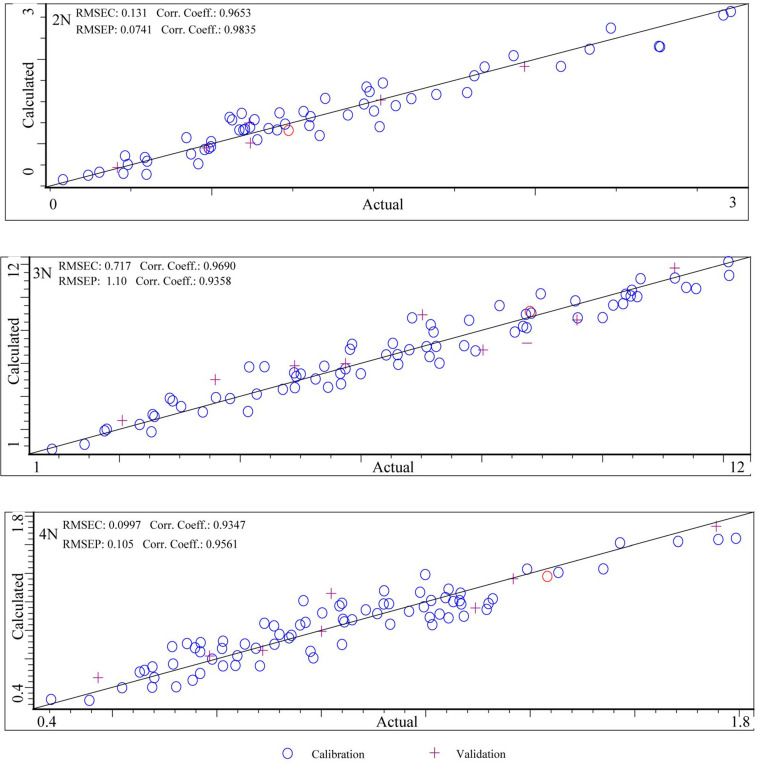
Correlation between the actual and predicted glycogen content values of different ploidy *C. gigas* using the MN instrument. From top to bottom: diploid, triploid, and tetraploid.

**Figure 8:**
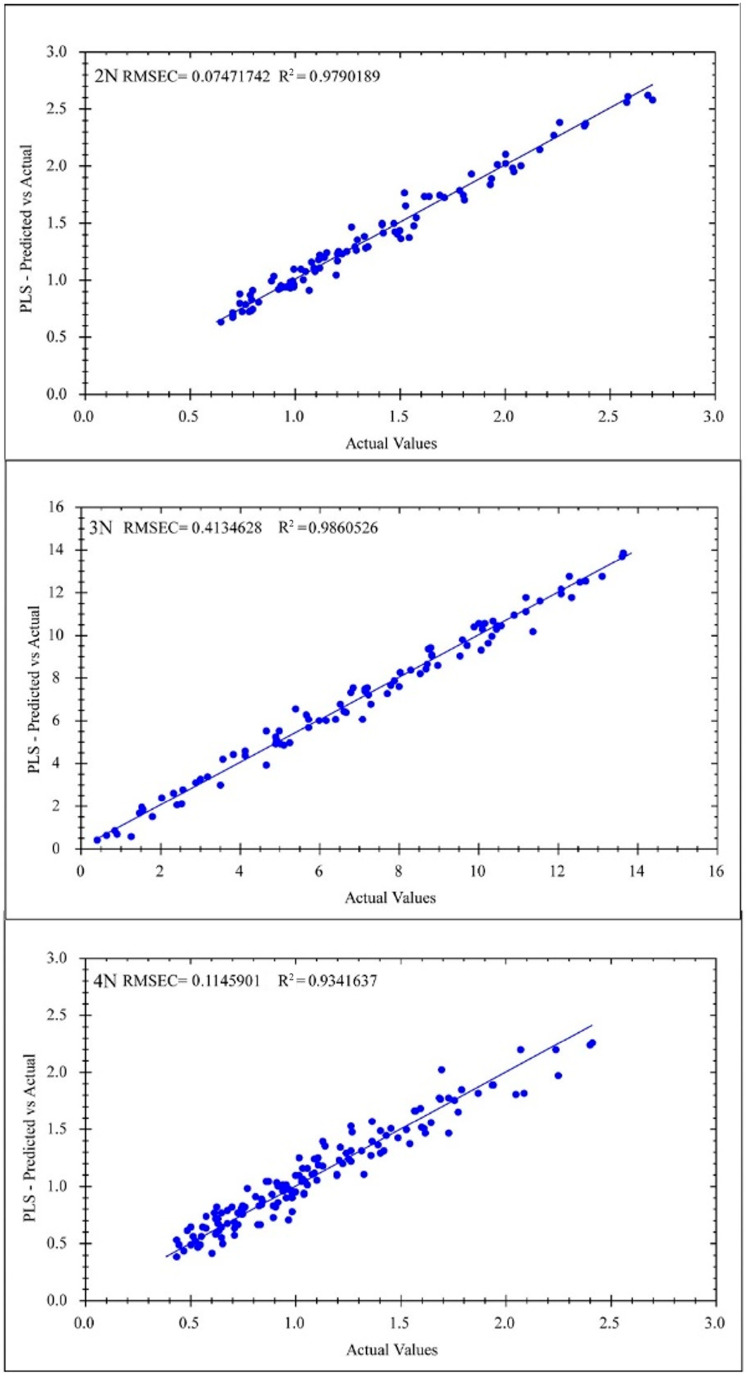
Correlation between the actual and predicted glycogen content values of different ploidy *C. gigas* using the MR instrument. From top to bottom: diploid, triploid, and tetraploid.

Two portable near-infrared spectrometers were employed to construct NIR models for glycogen content in *C. gigas* diploid, triploid, and tetraploid oysters. After determining the glycogen components in oysters, we optimized the spectral preprocessing methods. The quantitative models for glycogen content in oysters of different ploidies are shown in the table below. Six models were built with the MicroNIR 1700 spectrometer (Figure 7) and Micro PHAZIR RX spectrometer (Figure 8). There were some differences in the modeling effects of these two instruments. The specific values are shown in Table 5.

For the models built with the MicroNIR 1700 spectrometer: The RMSE_C_ and R_C_ for diploids were 0.131 and 0.965, respectively; the values for triploids were 0.717 and 0.969; the values for tetraploids were 0.100 and 0.935. These R_C_ values were all above 0.9, with the highest R_C_ value (0.969) observed in the triploid model. In internal cross-validation, the RMSE_CV_, R_CV_, and RPD_CV_ values of diploids were 0.158, 0.949, and 3.191, respectively, and the RMSE_CV_, R_CV_, and RPD_CV_ values for triploids were 1.170, 0.915, and 2.498. The RMSE_CV_, R_CV_, and RPD_CV_ values for tetraploids were 0.100, 0.902, and 2.310. All of the R_CV_ values were above 0.9, with the highest R_CV_ (0.949) observed in the diploid model. In external validation, the R_P_ values for different ploidy oyster models are all above 0.9, with the highest R_P_ value (0.984) observed in diploids. Additionally, all RPD_EV_ values were above 2, indicating high reliability of the model results. Better results were obtained with the short-range NIR range compared to the long-range NIR range. For the models built with the Micro PHAZIR RX spectrometer: In the calibration set, the RMSE_C_ and R_C_ for diploids were 0.075 and 0.979, respectively; for triploids, the values of RMSE_C_ and R_C_ were 0.413 and 0.986; and for tetraploids, the values of RMSE_C_ and R_C_ were 0.115 and 0.934. These R_C_ values were all above 0.9, with the best modeling effect observed in triploids (0.986). However, in internal cross-validation, the MP instrument did not show good modeling results. The values for diploids were 0.241, 0.781, and 2.240 for RMSE_CV_, R_CV_, and RPD_CV_, respectively; for triploids, the values were 1.106, 0.839, and 2.504; and for tetraploids, the values were 0.243, 0.717, and 1.851. R_CV_ did not show good validation results, with only the triploid model (0.839) exceeding 0.8. However, all RPD_CV_ values are above 1.4 [19,33], indicating relatively reliable model results.

#### 3.5.2. Spectral Characteristics and Instrument Differences

Glycogen mainly contains hydrogen groups with O–H bonds and C–H bonds, and the primary overtone absorption band of O–H bonds is around 1450 nm and 1900 nm, while the primary overtone absorption band of C–H bonds is around 1700 nm [34]. The MN instrument collects spectra in the wavelength range of 908–1676 nm, while the MR instrument collects near-infrared wavelengths in the range of 1624–2460 nm. Different wavelength ranges may affect the capture and interpretation of spectral characteristics of specific compounds. The differences in performance and wavelength range between the two instruments may result in varying spectral information collected at different bands, affecting the model’s performance. From the results of this study, the wavelength range of the Micro NIR 1700 may be more suitable for constructing the *C. gigas* glycogen model, leading to better results. Additionally, technical specifications, optical paths, and detector performance differences between the two instruments may affect their performance in spectral collection and processing. These technical differences could result in variations in signal-to-noise ratio, resolution, or sensitivity in certain wavelength ranges. Different preprocessing methods, such as smoothing, denoising, or standard normal variate, may have been employed for the two instruments in the study. The choice of preprocessing methods can significantly impact model performance. These factors may contribute to the MicroNIR 1700 spectrometer establishing a more stable and effective *C. gigas* glycogen near-infrared model. The use of the Micro PHAZIR RX handheld near-infrared spectrometer allows for real-time and on-site predictions, offering convenience with minimal environmental requirements.

#### 3.5.3. Biological Factors and Model Performance

Regarding the quantitative models for oysters of different ploidies, the triploid model performed better than the diploid and tetraploid models, demonstrating superior performance. Triploids, often sterile, tend to accumulate more glycogen compared to diploids and tetraploids, which can divert more energy towards reproduction. These differences in glycogen content can be attributed to the varying metabolic activities and physiological states of the different ploidy levels. The biological differences between triploid oysters, which include the characteristic of sterility, and diploid and tetraploid oysters may lead to variations in chemical composition and characteristics. The higher glycogen content in triploids may result in a data distribution more suitable for the modeling approach employed, making it easier for the model to identify and utilize these features for accurate classification or prediction. In addition, diploid and tetraploid oysters showed lower glycogen content and more variability in their spectral characteristics, which could lead to less accurate models. The diploid oysters’ active reproductive state and the tetraploid oysters’ genetic complexity might contribute to this variability. Different analytical instruments or techniques may have different sensitivities to specific spectral ranges or chemical compositions. In this context, the Micro NIR 1700 spectrometer may be more adept at capturing spectral signals related to glycogen content, which could be more pronounced in triploid samples. The combined impact of these factors may contribute to the superior performance of the triploid model in the quantitative model. During the process of construction of the NIR model and analysis, the biological differences and sample characteristics between different ploidies should be given careful consideration to optimize the modeling process. 

Wang et al. utilized a large-scale Fourier near-infrared spectrometer to analyze the meat composition traits, including protein, fat, moisture, and glycogen with diploid *C. gigas* [35]. Huang et al. conducted a similar analysis with *C. angulate* [36]. While these studies demonstrated effective analysis, their practical application is limited by the size and complexity of the large-scale instruments. In contrast, our study focuses on the use of portable NIR spectroscopy for analysis, offering greater portability and practicality in the field. Our results indicate that portable devices perform comparably to large-scale instruments in terms of analysis performance, and are more flexible and convenient for practical applications. This suggests a promising future for portable NIR spectroscopy in the genetic breeding field of polyploid oyster analysis.

**Table 5:** Indicators for modelling quantitative models for different ploidy samples.

Instruments	Ploidies	Calibration Sets		Validation Sets					
				Internal Cross-Validation			External Validation		
		RMSE_C_	R_C_	RMSE_CV_	R_CV_	RPD_CV_	RMSE_P_	R_P_	RPD_EV_
MicroNIR 1700	2N	0.131	0.965	0.158	0.949	3.191	0.074	0.984	2.240
	3N	0.717	0.969	1.170	0.915	2.498	1.100	0.936	2.780
	4N	0.100	0.935	0.122	0.902	2.310	0.105	0.956	3.565
Micro PHAZIR RX	2N	0.075	0.979	0.241	0.781	2.240	-	-	-
	3N	0.413	0.986	1.106	0.839	2.504	-	-	-
	4N	0.115	0.934	0.243	0.717	1.851	-	-	-

## 4. Conclusions

Sample analysis using a portable NIR spectrometer has the advantage of being non-destructive and rapid, which is particularly important for economically valuable and vulnerable biological samples such as oysters. This method protects the integrity of the sample while providing immediate analytical results. This study successfully employed two portable NIR spectrometers operating in distinct spectral ranges to acquire NIR spectra of the gonadal tissues of *C. gigas*. Quantitative models for glycogen in tetraploid *C. gigas* were established for the first time, as well as models for diploid and triploid oysters. Both instruments demonstrated satisfactory performance, and the cross-validation correlation coefficients (R_CV_) and cross-validation relative predictive errors (RPD_CV_) for the glycogen models revealed promising results. These findings emphasized the feasibility of predicting glycogen content in *C. gigas* using portable spectrometers, with the MicroNIR 1700 instrument particularly standing out for effective prediction of glycogen components in different ploidy oyster samples. Qualitative models were also established for different ploidies and genders of *C. gigas*. The qualitative model in this study demonstrated high performance in gender identification, with correct rates of 95% for diploids and 91.9% for tetraploids using the 908–1676 nm wavelength instrument. The 1624–2460 nm wavelength instrument successfully separated genders in diploid and tetraploid qualitative models, achieving correct identification rates of 100%. However, convincing results for different ploidies were not obtained. Through qualitative modelling, the study achieved high accuracy in gender identification, which has important applications for genetic breeding and population management of oysters. Especially in commercial farming, gender identification is essential for improving reproductive efficiency and optimizing production strategies. In conclusion, this study provides valuable insights for *C. gigas* breeding decisions based on meat quality traits, particularly with the establishment of the first glycogen model for tetraploid *C. gigas* using portable NIR spectroscopy. The successful application of portable NIR spectrometers provides new ideas for non-destructive and efficient evaluation of oyster quality. Both the short-range (MN) and long-range (MP) NIR spectral ranges provided valuable information, with each offering unique insights. Using both spectral ranges together as complementary techniques can enhance the accuracy and robustness of glycogen quantification and gender identification. This combined approach holds significant potential for improving non-destructive and efficient evaluation methods in oyster aquaculture and selective breeding practices.

## Figures and Tables

**Table 1:** Composition of Glycogen Content in Calibration and Validation Sets. N: number of samples.

NIR Instruments	Ploidy	Calibration Sets			External Validation Sets		
		Average	Range	N	Average	Range	N ^a^
MicroNIR 1700	2N	1.34 ± 0.50	0.54–2.60	56	1.24 ± 0.45	0.71–1.97	6
	3N	6.63 ± 2.92	0.89–12.09	70	6.51 ± 3.06	2.05–11.19	8
	4N	1.03 ± 0.28	0.48–1.79	78	1.08 ± 0.37	0.57–1.76	8
Micro PHAZIR RX	2N	1.25 ± 0.54	0.40–2.71	115	-	-	-
	3N	7.14 ± 3.52	0.41–13.63	93	-	-	-
	4N	1.08 ± 0.45	0.43–2.41	145	-	-	-

^a^ Represents number of samples.

**Table 2:** Preprocessing Methods and Principal Component Factors for Quantitative Models of Different Ploidies.

NIR Instruments	2N		3N		4N	
	Pretreatment ^b^	Principal Component	Pretreatment	Principal Component	Pretreatment	Principal Component
MicroNIR 1700	SD, NDF (5, 5)	8	SD, NS	8	SP, SG (7, 3)	9
Micro PHAZIR RX	SG, NR	20	SG, NR	20	SG	20

^b^ Represents the preprocessing method used by the model. SD: second derivative, NDF: Norris derivative filter, SG: Savitzky–Golay filter, NR: Normalize Range, NS: no smoothing, SP: original spectra.

**Table 3:** Preprocessing Methods, Principal Component Factors, and Performance Parameters for Three Qualitative Models of the Micro NIR 1700 Near-Infrared Spectrometer.

Models ^c^	Pretreatment	PC	Cumulative Contribution Rate	Performance Index	Accurately Judged or Not
2N-M, 2N-F	SD, NDF (5, 5)	10	99.90	91.6	Yes
2N, 3N, 4N	SG (7, 3)	10	99.95	83.1	No
4N-M, 4N-F	FD	10	99.97	95.0	Yes

^c^ Represents the sample used for qualitative modelling. 2N-M: diploid males, 2N-F: diploid females, 4N-M: tetraploid males, 4N-F: tetraploid males, FD: first derivative.

**Table 4:** Preprocessing Methods and Performance Parameters for Three Qualitative Models of the Micro PHAZIR RX Near-Infrared Spectrometer. AUC: area under curve.

Models	Pretreatment	Correctly Identified	AUC ^d^	Accurately Judged or Not
2N-M, 2N-F	SP	100%	0.922	Yes
2N, 3N, 4N	NR	81.17%	0.426	No
4N-M, 4N-F	SG, NR	100%	0.811	Yes

^d^ Represents the area under the ROC curve bounded by the axes.

## Data Availability

The original contributions presented in the study are included in the article, further inquiries can be directed to the corresponding author.
